# Normative nasalance scores in Chilean adults

**DOI:** 10.1590/2317-1782/20212021152

**Published:** 2022-03-25

**Authors:** Felipe Inostroza-Allende, Mirta Palomares-Aguilera, Matías Gonzalez Jara, Camilo Quezada Gaponov, Carlos Giugliano Villarroel, María Inés Pegoraro-Krook

**Affiliations:** 1 Departamento de Fonoaudiología, Universidad de Chile - Santiago, Chile.; 2 Fundación Gantz - Hospital del Niño con Fisura - Santiago, Chile.; 3 Smile Train - South American Medical Advisory Council – SAMAC - Santiago, Chile.; 4 Escuela de Fonoaudiología, Sede Santiago, Facultad de Salud, Universidad Santo Tomás - Santiago, Chile.; 5 Universidad de los Andes - Santiago, Chile.; 6 Unidad de Cirugía Plástica, Servicio de Cirugía, Clínica Alemana - Santiago, Chile.; 7 Departamento de Fonoaudiologia, Faculdade de Odontologia de Bauru, Universidade de São Paulo – USP - Bauru (SP), Brasil.; 8 Hospital de Reabilitação de Anomalias Craniofaciais, Universidade de São Paulo – USP - Bauru (SP), Brasil.

**Keywords:** Nasalance, Velopharyngeal Insufficiency, Cleft Palate, Speech and Language Pathology, Speech, es, Nasalancia, Insuficiencia Velofaríngea, Fisura del Paladar, Patología del Habla y Lenguaje, Habla

## Abstract

**Purpose:**

The present study is aimed towards determining and comparing normative nasalance scores in Chilean Spanish-speaking adult men and women.

**Methods:**

40 women (age range 18 to 35, X = 25.79, SD = 5.83) and 36 men (age range 18 to 35, X = 26.45, SD = 4.08) were invited to participate, all of them without any previous speech therapy, neurological pathologies, intellectual deficits, hearing loss, syndromes, or other diagnosed pathologies that could impact speech production.

A study of proper velopharyngeal function was performed, using a perceptual resonance evaluation. Nasalance was determined using a model 6450 Nasometer, during the reading of three standardized speech samples in Spanish: a nasal passage (NP), an oronasal passage (ONP), and an oral passage (OP). Also, the nasalance distance was calculated. Genders were compared using Wilcoxon tests for independent samples.

**Results:**

The NP presented the highest percentage of nasalance, with 52.13% (± 4.73), followed by the ONP with 25.38% (± 3.7), and finally the OP, which presented the lowest value of 14.15% (± 5.03). Meanwhile, nasalance distance was 37.98% (± 5.32). Finally, no significant differences were observed when comparing the nasalance between genders (p >0.05).

**Conclusion:**

The nasalance values obtained were similar to those observed for other Spanish speakers. Also, male and women showed similar scores. The results of this study are a contribution to the indirect assessment of velopharyngeal function in Chilean adults.

## RESUMEN

**Objetivo:**

El presente estudio tiene como objetivo determinar y comparar puntajes normativos de nasalancia en hombres y mujeres chilenos de habla hispana.

**Método:**

Participaron 40 mujeres (rango de edad de 18 a 35, X = 25.79, DE = 5.83) y 36 hombres (rango de edad de 18 a 35, X = 26.45, DE = 4.08), todos sin ningún antecedente de tratamiento fonoaudiológico, patologías neurológicas, déficit intelectual, hipoacusia, síndromes u otras patologías diagnosticadas que podrían afectar la producción del habla. La adecuada función velofaríngea fue determinada en vivo a través de una evaluación perceptual de la resonancia. La nasalancia se determinó utilizando un Nasometer modelo 6450, durante la lectura de tres muestras de habla estandarizadas en español: un párrafo nasal (PN), un párrafo oronasal (PON) y un párrafo oral (PO). Además, se calculó la distancia de nasalancia. La comparación entre ambos sexos fue realizada con el test de Wilcoxon para muestras independientes.

**Resultados:**

El PN presentó el mayor porcentaje de nasalancia con 52.13% (± 4.73), seguido del PON con 25.38% (± 3.7), y finalmente el PO, que presentó el valor más bajo de 14.15% (± 5.03). En tanto, la distancia de nasalancia fue del 37.98% (± 5.32). Por último, no se observaron diferencias significativas al comparar entre ambos sexos (p> 0.05).

**Conclusión:**

Los valores de nasalancia obtenidos fueron similares a los observados para otros hispanohablantes. Además, hombres y mujeres mostraron puntuaciones similares. Los resultados de este estudio son una contribución a la evaluación indirecta de la función velofaríngea en adultos chilenos.

## INTRODUCTION

A proper function of the velopharyngeal mechanism (VPM) or velopharyngeal sphincter has a great impact on the separation of oral and nasal cavities during the performance of diverse functions, such as speech, swallowing, and blowing^(1)^. During speech, the synergistic action of the soft palate and pharyngeal walls is essential to obtain a balanced oronasal resonance and to generate adequate levels of intraoral pressure during the production of oral sounds^(2)^. When complete velopharyngeal closure does not occur during the production of oral sounds, part of the voiced airstream is diverted towards the nasal cavity, affecting speech production in different ways. Due to this, excess acoustic energy in the nasal cavity alters the resonance’s balance^(3)^.

Nasality is known as the auditory perception of the nasal component of speech^(4)^, which may become altered due to conditions such as cleft palate, head and neck cancer, cerebrovascular accidents, and basal ganglia dysfunction, among others^(5)^. Cleft palate is one of the most frequent causes of increased nasality, with structural factors generating a failure in velopharyngeal closure, a condition known as velopharyngeal insufficiency (VPI). The primary surgical correction of the palate prioritizes restoring anatomical and functional conditions granting adequate velopharyngeal closure. Despite this, 15% to 25% of individuals with a cleft palate keep displaying VPI symptoms after primary palate surgery^(6,7)^. In such cases, an adequate assessment of the velopharyngeal function becomes necessary.

The gold standard to assess speech disorders related to velopharyngeal dysfunction (VPD) and cleft palate is perceptual evaluation conducted by a Speech Pathologist^(3)^. This assessment consists of detecting and characterizing the presence of hypernasality, hyponasality, audible nasal air emission, and/or nasal turbulence^(8)^.

The evaluation of velopharyngeal function during speech must be complemented with direct measures, involving methods such as flexible nasal endoscopy and multiview videofluoroscopy, and indirect measures such as spectral analysis, aerodynamic measurements and nasometry, among others. Of all these methods, nasometry is the most used indirect diagnostic tool when detecting abnormalities in resonance^(9)^.

The first instrument-based nasality standardization took place in 1970, with a machine called TONAR (The Oral Nasal Acoustic Ratio), which used psychophysical techniques to compare sound pressure levels between sounds emitted from the mouth and nose^(4)^. Different companies currently market nasometry equipment, with Nasometer, NasalView, and Oronasal System^(10)^ being some of the most common. They all estimate speech resonance by measuring nasalance, which corresponds to the relative amount of acoustic energy emitted from the nasal cavity during speech. Different authors have shown that nasalance scores effectively correlate with the perceptual judgment of nasality^(9,11,12)^. It is worth mentioning, that nasalance scores obtained among subjects with VPD should be compared with reference values observed for subjects with adequate velopharyngeal function^(11)^. To make this comparison, assessments of nasalance during the production of standardized speech samples have been implemented, which were composed predominantly of oral and nasal consonants to diagnose hypernasality and hyponasality, respectively. Also, an oronasal sample is evaluated, which contains a proportion of nasal and oral consonants comparable to that observed in a conversational speech sample^(12)^.

Reference values have been obtained in different languages to evaluate the velopharyngeal function according to country. The literature reports normative data for Brazilian Portuguese^(13)^, Australian English^(14)^, Flemish^(15)^, Korean^(16)^, Irish^(17)^, Japanese^(18)^, and Canadian English and French speakers^(19)^, among other languages. As for Spanish, studies have been conducted with participants from Puerto Rico^(12)^, México^(20)^, Colombia^(21)^ and Spain^(22)^. Also, because of structural differences in the velopharyngeal valve between men and women, some studies have analyzed the variations in nasalance between genders, reaching different conclusions^(14-16,18)^. Although the balance of high vowels versus low vowels in different languages might explain the difference in nasalance scores, the phonemic composition of a particular passage may also contribute to variation among the results. Some studies point out, that passages with more high vowels have higher nasalance scores than those with lower vowels, regardless of the language^(23,24)^.

Since the percentage of nasalance varies depending on particular dialects and/or languages, normative scores have to determined by evaluating speakers with no alterations in the VPM and speech, in each given region or country ^(15,17,18)^. Currently there is no comparison between countries. There are no previous normative nasalance scores for Chilean adult speakers. Thus, the present study aims at describing the nasalance scores of a group of Chilean adults of both genders with a proper adequate velopharyngeal function, and at comparing the nasalance between the sampled men and women.

## METHODS

This study was conducted in the Speech Therapy Department of “Fundación Gantz, Hospital del Niño con Fisura”, located at El Lazo 8545, Pudahuel, Santiago, Chile. All subjects underwent a nasometric speech evaluation (nasometry). Each session lasted approximately 30 minutes. This research was approved by the Ethics Committee for Research in Human Beings of the Facultad de Medicina, Universidad de Chile, number N° 208-2018. All individuals involved signed an Informed Consent Form prior to study commencement.

### Participants

Seventy-six native Spanish-speaking Chilean adults with adequate velopharyngeal function were evaluated and absence of dialect variation. Forty women (age range 18 to 35, X = 25.79, SD = 5.83) and 36 men (age range 18 to 35, X = 26.45, SD = 4.08). Participants were selected from the population of professionals, staff, and patients’ attendants at Fundación Gantz. No participants had a history of speech disorders, neurological pathologies, intellectual deficits, hearing loss, syndromes, or other congenital or acquired anomalies that affect speech production, as determined by the perceptual assessment of a conversational speech sample, carried out by a speech therapist with experience in evaluating patients with velopharyngeal dysfunction. On the day of the evaluation, all the participants had nasal patency in both nostrils, determined with a Glatzel mirror.

### Equipment and recording procedures

Nasalance (the acoustic correlation of nasality) was determined using a model 6450 Nasometer (Kay Elemetrics Corp., Lincoln Park, NJ). This equipment consists of two microphones, positioned on each side of a sound separation plate, which is placed above the individual's upper lip. The upper microphone captures the nasal acoustic signal and the lower microphone the oral acoustic signal during the production of the speech samples. Both signals are then filtered, digitalized by electronic software, and processed by the equipment's microcomputer. The nasalance score calculated by the program corresponds to the relative amount of nasal acoustic energy in speech, that is to say, the numerical ratio between the amount of nasal acoustic energy and the total acoustic energy (sum of nasal and oral acoustic energy), multiplied by 100. The system was calibrated daily, using a built-in sound generator source. Participants were asked to read the written stimuli at a comfortable pitch and loudness, starting with the oral passage, nasal passage and oronasal passage in a quiet room.

### Written stimuli

The assessment was conducted while participants read three passages^(12,25)^. The nasal passage El mono Memo (The monkey Memo)^(25)^, with 54% nasal consonants (Appendix A); the oronasal passage La oveja (The sheep)^(12)^, with 18% nasal consonants (Appendix B); and the oral passage Ella es Carla (She is Carla)^(25)^, which does not contain any nasal consonants (Appendix C). Three measurements were obtained from each passage for each participant, from which the average nasalance scores and their respective standard deviations (SD) were calculated. Besides, the mean and SD of the nasalance distance, defined as the quotient: oral passage / nasal passage, was calculated because it is an important complement to the mean values, which has been previously reported in other studies^(12,15,16,20)^.

### Statistical analysis of the data

The statistical analysis was conducted using the statistical software R (version 3.4.4, The R Foundation for Statistical Computing, Vienna, Austria). The obtained results were organized in tables showing measures of position (mean) and variability (standard deviation). To compare the nasalance results obtained between men and women, the Mann-Whitney U test was used. Alpha level was set at 0.05.

## RESULTS

Table 1 shows the mean and standard deviation (± SD), minimum and maximum score of nasalance and the nasalance distance obtained for all participants. The nasal passage presents the highest percentage of nasalance, 52.13% (± 4.73), followed by the oronasal passage with 25.38% (± 3.70), and the oral passage, which presents the lowest value of nasalance, at 14.15% (±5.03).

**Table 1:** Mean (±SD), minimum and maximum scores of nasalance and nasalance distance obtained for all participants

	n	Mean (±SD)	Minimum	Maximum
Nasal Passage	76	52.13 (±4.73)	37	60
Oronasal Passage	76	25.38 (±3.7)	17.5	34.66
Oral Passage	76	14.15 (±5.03)	8	28
Nasalance Distance	76	37.98 (±5.32)	24.5	49

Table 2 presents and compares nasalance scores obtained for both genders. Even though higher percentages were observed in all passages for females, these differences were not statistically significant (p >0.05).

**Table 2:** Mean nasalance scores (%) and SD for nasal, oronasal, oral passages and nasalance distance by Chilean speakers, according to gender

	Mean (±SD) (females, n = 40)	Mean (±SD) (males, n = 36)	P-value
Nasal Passage	53.42 (±4.27)	50.6 (±4.9)	0.092
Oronasal Passage	26 (±3.31)	24.65 (±4.07)	0.215
Oral Passage	15.48 (±5.95)	12.57 (±3.13)	0.197
Nasalance Distance	37.94 (±5.75)	38.02 (±4.91)	0.723

p<0.05

## DISCUSSION

The present study describes nasalance scores obtained during the production of three passages (oral, nasal, and oronasal) in Chilean Spanish-speaking adults of both genders with adequate velopharyngeal function. Additionally, values for men and women were compared.

As reported for other languages^(15,16)^, the nasalance scores obtained from this study's participants showed a progressive decrease in the percentage of nasal energy as the number of nasal consonants in each passage decreases (nasal, oronasal, and oral, respectively).

The mean values obtained in this study differ from other Spanish-speakers. Adult women from Puerto Rico showed a mean nasalance score of 62.07% in a group of nasal sentences different from those used in this investigation, a nasalance score of 36.02% for the oronasal passage La oveja, and a percentage of 21.95% for a different oral passage without nasal consonants^(12)^. The values from Puerto Rico were higher than those observed in the women present in this study, with a difference of 8 to 10% in nasalance scores.

Nichols (1999) reported that Mexican Spanish-speaking adults and children of both genders have a mean nasalance score of 17.02% for oral sentences without nasal consonants, and a mean nasalance score of 55.28% for nasal sentences, where 20% of the sounds were nasal consonants^(20)^, being 3% less than the nasalancia values ​​of this study.

The nasalance distance obtained in Chilean Spanish-speakers (37.98%) was 2.14% lower than the one observed in the speakers from Puerto Rico (40.12%)^(12)^, and 0.28% lower than the score of speakers from Mexico (38.26%)^(20)^. These values in Spanish-speakers from different countries are similar to the value of Irish-speaking children (37%)^(17)^ but lower than those observed in Korean adults (50.08%)^(16)^, Australian children (46.50%)^(14)^, and young Flemish speakers (44.90%)^(15)^. These differences may be due to differences in language, age, sampling, among others.

A study of normative nasalance performed in Colombia evaluated a series of 13 words conformed mainly by nasal consonants. This research reported a nasalance score of 39.70% in children of both genders between 3 and 5 years of age^(21)^. The nasalance measurements found in these children are lower than those found in the present study. In this regard, nasal resonance can be lower in children when compared to adults, this could be explained by the presence of a shorter vocal tract and greater size of the nasopharynx's obstructive structures (such as adenoid) in children^(26)^.

Various factors can influence the oronasal resonance in speakers with adequate velopharyngeal function, such as the type of vowels^(27)^, language^(15)^, regional accent^(11)^, length of the stimulus^(28)^, small changes in tongue height during the articulation of vowels, and variations in prosody^(5)^, among others, being able to influence the results obtained. Additionally, it has been reported that other factors related to the nasometry procedure modify the obtained nasalance score. Watterson (2005) showed that head position significantly modifies the obtained nasalance score^(29)^. Also, the Nasometer model could be a factor influencing the results. Awan and Virani (2013) compared the nasalance score obtained using the 6200 and 6400 models, reporting significant differences in results in the same speech samples^(19)^. In this regard, the Nasometer model in previous studies carried out on Spanish-speakers has been the 6200, and the present study utilized the 6450 model. A previous study found a difference in nasalance of up to 8% between the 6200 and 6450 model^(30)^. This difference is similar to that observed between women’s values from Puerto Rico and the present study’s results^(12)^.

Regarding gender, this study found no statistically significant differences between genders. Previous studies observed no significant differences between men's and women's scores^(14,16,18)^. These results are contrary to other reports, where the authors recorded higher nasalance scores of women in the nasal, oronasal, and oral passages^(15)^.

Finally, this study's findings show that nasometry is a tool that can be used for indirect instrumental assessment of velopharyngeal function during speech. However, in future research one should increase the sample size, expand the age range to be studied, and incorporate the use of new speech samples such as sentences and lists of words and syllables. These can be used in subjects who cannot read passages, mainly young children undergoing primary palatoplasty and surgeries for VPI^(13)^.

## CONCLUSION

This study presents the first normative values of nasalance for Chilean Spanish speaking adults, where the percentages obtained by men and women did not differ significantly. The results of the oral, nasal, and oronasal passages will be useful for the indirect assessment of the velopharyngeal mechanism in interdisciplinary care centers for patients with velopharyngeal dysfunction throughout Chile.

## Appendix A Nasal Passage^(25)^

**Table:**

El mono Memo (The Monkey Memo)
Una mañana mi mamá con mi hermana compraron mucho maní para que coman los monos en el campo. El mono más bonito es Memo. Él tiene un moño armado con mucho amor por su dueño. Memo come más bananas que maní.

## Appendix B Oronasal Passage^(12)^

**Table:**

La oveja (The sheep)
La oveja es un animal herbívoro. Se alimenta de hierba. Habita en todos los climas. Es un animal manso y resistente. Se mueve constantemente, pero es dócil a la voz del pastor y se deja guiar por los perros. Todo es útil en la oveja. La lana sirve para fabricar vestidos, mantas y alfombras. La piel se usa para abrigos y objetos de adorno. Su carne es sabrosa y con su leche se hacen quesos.

## Appendix C Oral Passage^(25)^

**Table:**

Ella es Carla (She is Carla)
Ella es Carla. Acaba de despertar. Va a la ducha. Luego se seca y se viste para ir al colegio. Bebe su leche y luego se sube al auto de su papá. Él la lleva al colegio para que estudie y juegue, a las cuatro su papá la lleva a su casa. Hace sus tareas y a las ocho se va a acostar.
